# Phytotoxicity Assessment of *Solanum lycopersicum* L. Seedlings Moderately Irrigated with Non-Thermal Plasma Treated Water Containing Sulfamethoxazole

**DOI:** 10.3390/plants14091277

**Published:** 2025-04-22

**Authors:** Marius Cicirma, Marius Dumitru, Sergiu Emil Georgescu, Aurora Neagoe

**Affiliations:** 1 Faculty of Biology, University of Bucharest, Splaiul Independenței, No. 91-95, 050095 Bucharest, Romania; cicirma.marius@bio.unibuc.ro (M.C.); auroradaniela.neagoe@g.unibuc.ro (A.N.); 2 National Institute for Lasers, Plasma and Radiation Physics, Atomistilor Str., No. 409, 077125 Magurele, Romania; marius.dumitru@inflpr.ro; 3 “Dan Manoleli” Research Centre for Ecological Services—CESEC and “Dimitrie Brândză” Botanical Garden, University of Bucharest, Aleea Portocalelor No. 1-3, Sector 6, 060101 Bucharest, Romania; 4 Research Institute of the University of Bucharest—ICUB, Panduri Road, No. 90-92, 050663 Bucharest, Romania

**Keywords:** phytotoxicity, tomato, soil irrigation, plasma-activated water, non-thermal plasma, advance oxidation process, treated water, sulfamethoxazole, antibiotic

## Abstract

Contamination of agricultural ecosystems with antibiotics including sulfamethoxazole (SMX) can create favorable conditions to increase bacterial abundance in soil with antibiotic-resistant genes and can also affect plants. The aim of this research was to assess the phytotoxicity of tomato after irrigation with SMX degraded in 20 min using the non-thermal plasma-ozonation technique (T20). To achieve this, two experiments were performed at the scales of Petri dishes and pots using *Solanum lycopersicum* L. species, cultivar Zaraza, subjected to irrigation treatments that were compared to a distilled water control. In plates, T20 solution improved root length and also seedling vigor indexes, but the germination index, germination speed, and biomass were slightly decreased. In soil, although T20 reduced the seedling root length, their growth was not inhibited (15.3%)**,** while in plates they exhibited a growth promotion effect with 90% more than the control. The physical–chemical and geochemical variables measured in the soil were suitable for crop characteristics and plant growth and showed statistically significant variations after harvesting. In T20-treated shoots, compared to SMX, better results were obtained for their length, assimilatory pigments, and biomass, thus selectively reducing the tomato seedling phytotoxicity depending on the endpoints, type of control, and growth methods tested.

## 1. Introduction

Polluted waters containing pharmaceuticals that are used for irrigation in agriculture can facilitate the accumulation of drugs into the soil and further translocation in plants at various concentrations [1,2,3,4]. Antibiotics such as SMX or its primary transformation product N4-acetyl-sulfamethoxazole (Ac-SMX) have been shown to be globally persistent both in aquatic environments [5,6] as well as in crops irrigated with reclaimed wastewaters [7]. These are lately affecting plants, fungal communities, and the natural microbial abundance. Antibiotics were shown to be ubiquitous in the surface water worldwide, in concentrations ranging from ng L^−1^ to μg L^−1^ [4,8] in scale, which represents a concern for potential adverse effects on non-target species [9] or to develop some antibiotic-resistant genes [5,10,11,12]. The degradation of the sulfonamides antibiotic class to which SMX belongs, and its plant-associated metabolism or phytotoxicity at different concentrations, were shown in some other studies but are still limited to a few species [1,11,13,14,15]. The uptake mechanisms depend on many factors associated with chemical composition, properties, or ionization of the pharmaceutical compound, as well as the biology of the plant species, growing medium, or exposure time period [2,16,17,18].

Like other sulfonamides, SMX disrupts the folate biosynthetic pathway in bacteria, raising concerns over non-target toxicity, such as plants [19]. In some of the studies in which SMX was added to plant growing media, different levels of bioaccumulation occurred even in tomato fruits [18,20]. Despite the use of various bioremediation methods to improve the quality of edible vegetables or to reduce the pharma pollution [21,22], plant phytotoxicity is induced by vegetable-dependent mechanisms that can influence the level of bioaccumulation [23]. However, pharmacokinetics modeling approaches indicate that roots are primarily affected, followed by stems and leaves by translocation, drug-dependently [24]. For example, the magnitude of SMX bioaccumulation quantified in tomato plants varied at different levels, being influenced by factors such as uptake rate, metabolism, and translocation capacity in tissues [18,20]. In most cases, this is directly related to the accumulation within the growing medium when reclaimed wastewater or organic amendments of animal origin are applied [7].

Since detoxification mechanisms and plant species tolerance to environmental pollution are mostly activated [25], the resulted acceptable daily intake (ADI) calculated for the sulfonamide class is mostly safe for human consumption. In some other studies, low-risk threats for such small drug concentrations were communicated [2], including tomatoes [7,18,26].

However, to avoid the presence of drugs within edible vegetables and a possible increase in antibiotic-resistant genes [27] in soil microbial communities [11], alternative and appropriate wastewater treatment is now being explored. For example, the advanced oxidation process technologies for wastewater treatments have been found to be an effective, new, and challenging complementary technique [28,29,30,31].

Wastewater treatment plants (WWTPs) can use various purification processes as primary (physical), secondary (biological), and tertiary (chemical) stages. The combination of these three methods of pollutants removal can also be applied to ensure acceptable water quality, depending on their investment facilities. Although some techniques and methods are broadely addressed [32,33] for water treatment, most of them are running at laboratory scales. Advanced oxidation processes (ozonation, Fenton process, non-thermal plasma, cold plasma, photo-catalysis, etc.) have been successfully applied for the degradation of a wide range of emerging organic contaminants in waters [30]. The non-thermal plasma (NTP) water treatment method has the advantage of generating highly reactive species [34] without the need for the addition of oxidants [35]. On the other hand, plasma in liquid media generates oxidizing species (O_3_, H_2_O_2_, ONOO-, etc.) that have the ability to degrade organic pollutants in water [36].

Moreover, advanced plasma applications have been achieved in research trials for seed treatment [37,38,39,40,41], as well as for water decontamination from various organic pollutants, including antibiotics [29,30,35].

However, many conventional WWTPs are not able to remove the low concentrations of persistant micro-pollutants, including pharmaceuticals. Urban wastewaters are important point sources for antibiotic pollution and antimicrobial resistance [30]. Treatment with non-thermal plasma (NTP) in combination with ultrafiltration (UF) membranes [42] was achieved together with many other reported operational designs. The removal rate of contaminants depends on a number of factors, such as their biodegradability, physicochemical properties, concentration, treatment process, operating parameters, etc. as shown in a review by Magureanu et al. (2021) [30].

**Figure 1:**
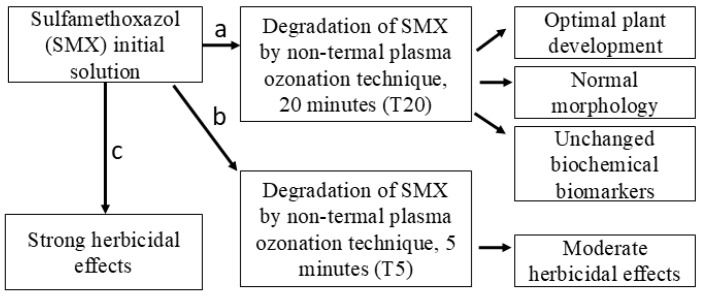
Schematic representation of key concepts for hypothesis testing.

The pharmaceutical compound SMX was selected for this study based on its risk to non-target species [43], as well as its known herbicidal mode of action in plants [19,44]. SMX has been degraded using controlled conditions of NTP [35,36,45]. However, knowledge about plasma–soil–plant–antibiotics interactions is scarce. For this purpose, the following hypothesis was tested: the SMX-free solution obtained as a result of its degradation by NTP after 20 min treatment (T20) will significantly reduce the overall plant toxicity in Petri dish and pot experiment scales, while the initial solution (SMX) and its 5 min partial treatment sample (T5) will induce strong and moderate herbicidal effects, respectively. A schematic key concept model for testing this hypothesis is presented in Figure 1.

## 2. Materials and Methods

### 2.1. Characterization of the Irrigation Solutions

In this study, six types of aqueous solutions were used for the irrigation of plants. These were distilled water (DW), tap water (TW), plasma-activated water (PAW) treated for 20 min in the ozonation system, sulfamethoxazole initial solution prepared in tap water (SMX), SMX treated with NTP for 20 min (T20), and SMX solution treated with NTP for 5 min (T5). DW ensures the physiological plant growth without interference from other factors, thus allowing experimental reproducibility.

The TW used (ApaNova, Bucharest, Romania) was characterized according to the official quality certificate publicly released by the supplier company for the distribution point. Blvd. Ion Mihalache was monitored during the period from 14 February to 6 March 2023, during the experimental time, and pH and electrical conductivity (EC) were additionally measured in our laboratory. TW mimics more realistic agricultural irrigation practices, improving the condition of plants through its content of dissolved salts and minerals, and is also suitable for optimal plasma discharge conditions above the liquid. The initial SMX (CAS 723-46-6, >98%, TCI) solution had a concentration of 0.25 mM, prepared using TW. It should be noted that the optimal discharge of plasma above the liquid requires a specific pH closer to that of tap water, which influences the competition for the OH radical formation in the degradation and ozonation reactions of organic compounds. Complete degradation of SMX was successfully achieved in previous studies by plasma exposure for 20 to 60 min, as reported by Bilea et al. [33]. In the current experiment, a time within the successful range (T20) was used, but an intermediate degradation time of 5 min (T5) was also considered. T5 was used to remove SMX by only 76%, so the effect of the remaining concentration of approximately 15 mg L^−1^ was followed by the evaluation of different plant variables. On the other hand, T5 contains, in addition to the basic compound, related reaction by-products that could induce moderate herbicidal effects [19]. The PAW was used to eliminate any potential plant response (+/−) induced by plasma discharges or possible SMX residues present in the solution after its treatment at T20. The entire ozonation system was previously described [35,45,46]. The technical preparation of the water treated by the NTP-ozonation system is time-consuming and limited to a running volume of 330 mL. All applied solutions were analyzed before irrigation in our laboratory (Table 1).

### 2.2. Experimental Set-Up

Two phytotoxicity tests were performed, one at the Petri dish scale and the second at the pot scale. In both experiments, the tomato seeds used were untreated for pests and diseases—the species *Solanum lycopersicum* L. cv Zaraza (Four Agro Bucharest, Romania). The two tests lasted 10 days in Petri dishes and 30 days in pots, respectively, from seed germination to cotyledon opening, and from sowing to seedlings. In order to uniformly assess plants’ development and their phytotoxicity, controlled growth conditions were maintained at 25/19 °C with a 16/8 h day/night cycle, light intensity of 5000 Lux, and 60% humidity. A daily randomized position of plates and pots in the growth chamber (Daihan LabTech Co., Ltd., Gyeonggi-do, Republic of Korea) was ensured.

#### 2.2.1. Petri Dishes Assay and Evaluated Endpoints

In the Petri dish scale experiment, we placed 20 tomato seeds in 3 replicates (*N* = 60) on 90 mm filter paper (Fisher Scientific, UK) in sterile plates (TPP) and 5 mL of each irrigation solution (DW, TW, PAW, T20, T5, and SMX). The plates were sealed with laboratory film (Parafilm, Bemis^®^, USA) and incubated for 10 days. Radicle emergence and root elongation were monitored from the 1st to the 8th day by ImageJ software, V 1.53. At the end of the experiment, roots and shoots were manually measured (day 10) and weighed (fresh and dry). Germination percentage: G = (*N*/A) × 100, where G is the germination percentage, *N* is the number of germinated seeds, and A is the number of seeds in the sample. Germination index: [G_*i*_ = (gs_1_/d_1_; gs_2_/d_2_…gs_10_/d_10_)], where gs_(x)_/d_(x)_ represents the number of germinated seeds divided by the number of respective days. The germination speed index (GSI) was calculated by daily counting of germinated seeds: GSI = Σ P*i*/D*i*, where GSI is the germination speed index, P*i* is the number of seeds germinated on the *i*th day, and D*i* is the number of days from the start of the test to the *i*th day. The germination speed was calculated as the vigor index I by length: $\sum_{i=1}^{10}Ni/Ti$, where *Ni* is the number of seeds germinated during the *i*th time interval and *Ti* is the time, in days, until the *i*th interval. The vigor index II was used for calculation by weight of the seedling dry biomass (milligrams per plant). Finally, the root inhibition effect: E_in_ = [(L_c_ − L_e_)/L_c_] × 100, where E_in_ is the inhibition effect (%), L_e_ is the average root length in the experiment (cm), L_c_ is the average root length in the DW control (cm) corresponding to 0% inhibition effect. The phytotoxic inhibition effect is considered when E_in_ is greater than 20%. Therefore, our recorded negative values were interpreted as representing the same percentage of root inhibition, allowing us to determine if a growth-promoting effect occurs [47,48,49].

#### 2.2.2. Pot Assay and Measured Biomarkers from Plants

The pot scale experiment was conducted to obtain a larger biomass for further investigations, and also to simulate the development of seedlings under more realistic field conditions [50]. Thus, the experiment was set up, consisting of 30 polyethylene pots (500 mL) containing six experimental treatments with 5 replicates each, named after the irrigation solution (as in the Petri dish experiment). In each pot, 12 tomato seeds were placed at a depth of 2 cm (*N* = 60 per treatment) in 450 g of moistened soil, based on its water holding capacity (WHC). The plants were irrigated daily to maintain the WHC with solutions prepared as described in Section 2.1 of this article. The experiment lasted 30 days, while the exact dates of the irrigation treatments (except DW) were on day 0 (at the installation of the experiment), days 1–15, 18–20, and on day 25 after sowing. In addition, for each treatment, an extra pot was supplemented for preliminary qualitative tests on the leaf and root surface microarchitecture. The mycorrhizal potential of the roots was also checked following soil inoculation with 10% fungi [51], using the *Rhizophagus irregularis* species as a mycorrhizal inoculum produced and sold by INOQ GmbH, Germany. This inoculum contained 210 spores per square centimeter, sequestered in 2 mm volcanic expanded clay particles.

Before harvesting the plants, stomatal conductance measurements were performed on the adaxial surface of the leaf [52,53] using a portable foliar porometer (Decagon SC-1, Decagon Devices Inc., WA, USA). The generated values were obtained at room temperature for two different tomato plants in each pot (2 individuals × five replicates), resulting in a total of *N* = 10 for each experimental treatment. Leaf surface microarchitecture, morphology, and stomatal aperture were investigated with a 15 kV scanning electron microscope (SEM): the FEI Inspect S50 model (FEI Company, Hillsboro, OR, USA). The vegetal material was initially gold-coated, followed by energy-dispersive X-ray spectroscopy (EDX) analyses for elemental spectrum detection. After harvesting, all plants were separated into roots and aboveground parts, and then, root and shoot morphometry was performed, following the recording of fresh biomass. The aboveground part of the plants was quickly rinsed with distilled water. After that, all plant material was frozen at −20 °C. Subsequently, the plant material was lyophilized (Martin Christ, Gefriertrocknungsanlagen GmbH, Germany), and the dry biomasses of roots and shoots were recorded, then ground in a stainless-steel mill equipped with a cooling system (IKA, 156 A11 basic, KA-Werke GmbH & Co. KG, Germany), and finally stored at −20 °C until processing.

The level of lipid peroxidation was measured in the dried roots and shoots of tomato seedlings by weighing 10 ± 0.1 mg and adding 3 mL of a solution containing 10% trichloroacetic acid and 0.25% thiobarbituric acid in ultrapure water [54]. Photosynthesis was investigated by quantifying the assimilatory pigments, namely chlorophylls and carotenoids, by weighing 30 ± 0.5 mg of vegetal material to which 4 mL of an extraction solution containing 80% acetone, 19.5% ultrapure water (18.2 MΩ·cm resistivity), and 0.5% ammonium hydroxide were added [55]. Microscopic observation of roots to check the colonization of *Rhizophagus irregularis* was performed on fresh root fragments until 2 cm long (one fragment from each individual plant grown in the additional pot introduced for this purpose). The fixing solution contained 45.85% ultrapure water, 45.85% (*v*/*v*) ethanol, 6% (*v*/*v*) formaldehyde, and 2.3% (*v*/*v*) acetic acid that was stored at 4 °C [56]. The histochemical staining steps were as follows: cleaning with 2% (*w*/*v*) KOH at 95 °C, rinsing with distilled water 3 times on a fine sieve, acidification with 2% HCl (*v*/*v*) for 30 min, and, finally, staining with 50% lactophenol blue solution (Merck KGaA, Darmstadt, Germany) [57]. Subsequently, the stained root fragments were transferred to slides with 100% glycerol and the fungal structure of the root segments was visualized by microscopy (Carl Zeiss Axio Imager 2, Jena, Germany).

#### 2.2.3. Description of the Selected Soil and Measured Variables

The reddish-brown soil used in the pot-scale experiment was sampled from the Moara Domneasca educational farm, University of Agronomic Sciences and Veterinary Medicine of Bucharest, Romania (geographical coordinates: 44°30′04″ N, 26°15′03″ E). This soil has been previously described [58] as loamy with medium-fine structure due to its high percentage of clay, especially in the upper part of the horizon. For this experiment, the soil was sampled from a depth of 0–20 cm, corresponding to the most biologically active layer. The samples were preliminary processed (manual removal of plant debris and any rocks, sieving through a 0.6 mm glass fiber sieve, homogenization, and drying at room temperature, away from sunlight). After preliminary processing, the soil was mixed into a single composite sample, then the following physical–chemical variables of the soil were measured: pH (H_2_O, 1:2.5 *v*/*v*) and EC (H_2_O, 1:5 *v*/*v*), for both, using a WTW 340i multiparameter system from Weinheim, Germany. The soil moisture was assessed after pre-drying the samples at 105 °C until reaching constant weight. Finally, analyses of mineral nitrogen forms (N-NH_4_^+^; N-NO_3_^−^; N-NO_2_^−^) and bioavailable phosphorus (P-PO_4_^3−^) analyses were performed. All N extractions were performed by weighing 20 g of wet soil, adding 100 mL of 0.2 M KCl, and shaking at 150 rpm for 1 h. For P, 5 g of soil with 0.5 M NaHCO_3_ was used, shacked at 150 rpm for 30 min. Then, all samples were filtered using medium-porosity filter paper and, finally, analyzed by spectrophotometric methods (CECIL Aquarius, Milton, Cambridge, UK). Ammonium was measured using a sodium salicylate solution, followed by a sodium nitroprusside solution that in the presence of sodium dichloroisocyanurate forms a green complex [59] spectrophotometrically [60]. Nitrate [61], and phosphate, were all measured [62]. After 30 days of draying, WHC was measured by the gravimetric method [63], loss on ignition (LOI) determining the organic matter lost at 600 °C [64], and macro- and microelements content of the soil (<2 mm mesh size) by using a handle X-ray fluorescence instrument (Thermo Scientific Niton GOLDD, Winchester, UK). Before starting the experiment, the soil was autoclaved twice for 30 min at 120 °C using a Raypa^®^ Steam Sterilizer (Barcelona, Spain). Except for WHC, all described variables were measured before sowing and after harvesting the plants [65,66].

### 2.3. Statistical Analysis

Graphs and data sets were generated and calculated using Origin^®^ 6.0 software and statistical differences of means were performed by analysis of variance (ANOVA), with one-way ANOVA against controls and between treatments, where the significance level (*p* ≤ 0.05) was marked with lowercase letters above the columns or occasionally with the symbol NS for not statistically significant.

## 3. Results

### 3.1. Seed Germination, Vigor Indexes, and Seedling Morphometry

**Figure 2:**
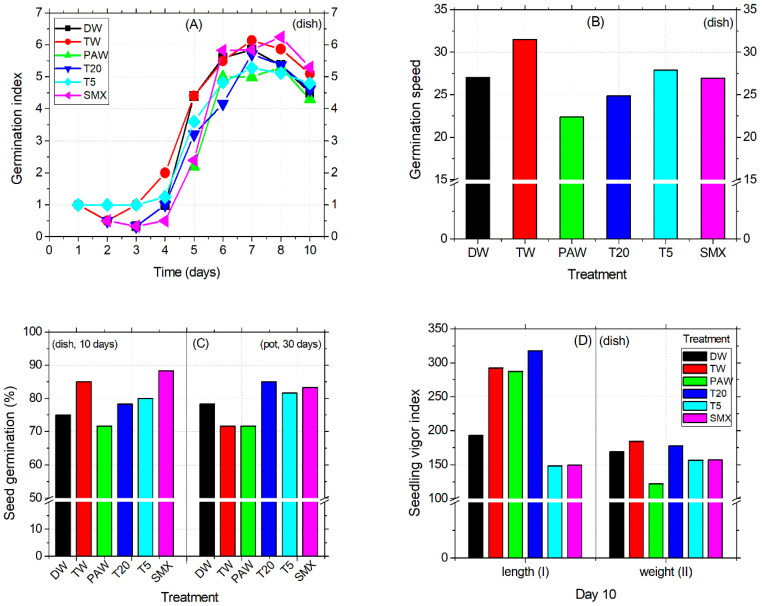
Germination index (**A**), germination speed (**B**), germination percentage (**C**), vigor index I and II (**D**).

The seed germination was first initiated by TW and T5 treatments after 24 h, followed by T20 and SMX on the second day. At this time, TW had decreased to half of the previous day, by 0.5 units, and then was further restored until day 3. DW treatment induced physiological germination after three days of sowing, while T5 remained constant during this time period. PAW treatment slowed down the process by 48 h more than DW. Next, on day 6, the number of germinated seeds immersed in the PAW solution exceeded that of the previous date, thus reaching an index almost equal to that of DW and TW (Figure 2A). The low radicle emergence performance of PAW-soaked seeds during observations at the middle exposure time was further supported at the end of the experiment by the vigor index II (by weight). Here, the range differences in PAW treatment compared to the other treatments were between 30 and 60 units, suggesting a low seed fitness for germination capacity under these experimental conditions (Figure 2D). In contrast, the vigor I index of PAW (by length) was as appropriate as TW and T20 (day 10), leading to a final percentage of seed germination that remained the lowest of all the tested treatments (Figure 2C). Overall, the speed of seed germination decreases in the following order: TW > T5 > DW > SMX > T20 > PAW, as shown in Figure 2B.

**Figure 3:**
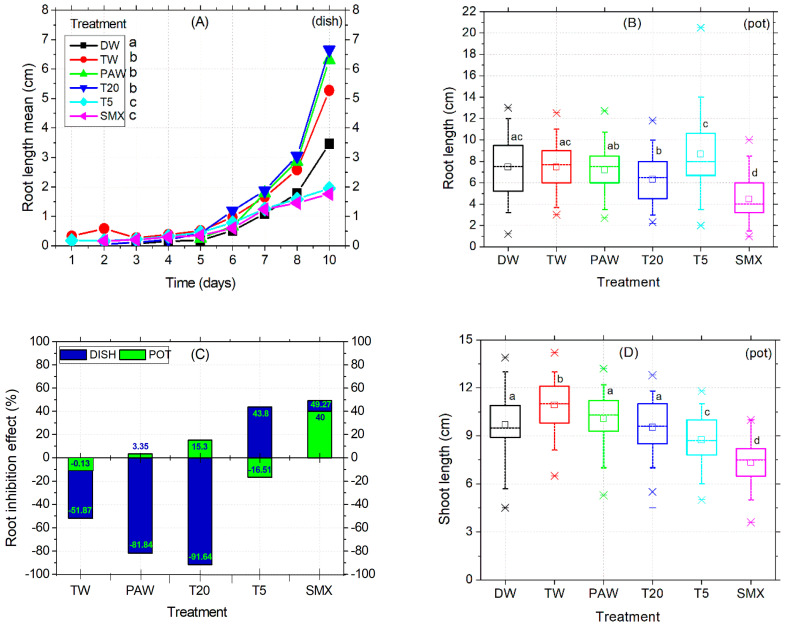
Root elongation timeline (**A**), root length (**B**), root inhibition effect (**C**), and shoot length (**D**). Statistical differences of means between treatments are marked with lowercase letters above the columns.

In the Petri dish assay, shoot length was not affected by the treatments applied. Instead, the early root growth response was enhanced by the TW, PAW, and T20 treatments compared to the DW control (Figure 3A) and confirmed by the absence of inhibitory effects (Figure 3C). The intermediary T5 treatment reduced the root size in the plates (Figure 3A), while in the pot assay, it significantly increased compared to PAW, T20, and SMX (Figure 3B, outlier). The PAW solution promoted almost double the root growth compared to the TW treatment, followed by T20, although the T20 solution still contained other persistent by-products after SMX degradation using the NTP-ozonation setup [35]. Overall, there were no significant differences between PAW and TW for the root length endpoint (Figure 3A). However, T20 did not achieved the root inhibition effect in the pot test, because the percentage is less than 20% (Figure 3C).

Conversely, SMX induced root growth inhibition in both the Petri dishes and pot assays twice above the 20% threshold (Figure 3C), thus maintaining its herbicidal properties over time. Shoot length differences for the DW control were significantly higher compared to T5 and SMX treatments but lower than the TW and similar to PAW and T20. The solvent control TW had the highest mean value among all treatments. In contrast, the initial SMX solution gave the lowest shoot sizes across all length averages, followed by T5 in the pot assay (Figure 3D). However, exposure of tomato cv. Zaraza seeds to the SMX solution significantly improved seed germination in a Petri dish (53 out of 60) during a ten-day assessment, compared to DW and PAW (Figure 2C), but the final dry root biomass had the lowest value among all treatments (0.46, Table 2). This is correlated with the lowest root length of SMX-treated seeds in the Petri dish (Figure 3A). In contrast, the total weight of shoots and roots treated with PAW was higher than the DW and TW controls, while T20 recorded the lowest values of all treatments. Table 2 presents data from composite samples (three replicates).

### 3.2. Plant Morpho-Physiological and Oxidative Non-Enzymatic Stress Responses

**Figure 4:**
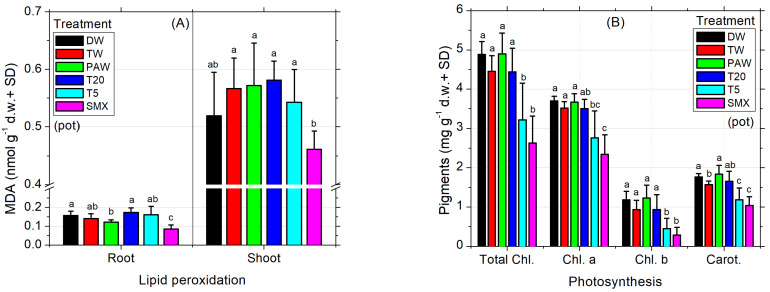

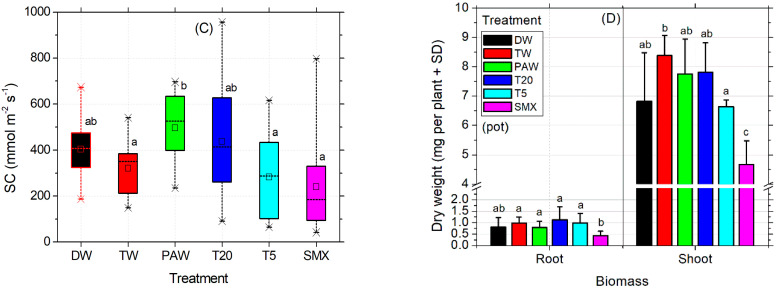
Lipid peroxidation (**A**), photosynthesis (**B**), stomatal conductance (**C**), and biomass (**D**). Statistical differences of means between treatments are marked with lowercase letters above the columns.

The MDA levels recorded in SMX-treated roots were the lowest compared to all treatments, while in shoots they were appropriate to the DW control (Figure 4A). T20 induced comparable values to all controls. Although the low level of MDA from SMX treatment seems beneficial for the plant, the photosynthesis was affected as the total chlorophyll (a and b) and carotenoids were strongly decreased compared to all treatments and controls, except the T5 solution (Figure 4B). Improvements of lipid peroxidation status compared to the absolute DW control by low MDA content are shown in the PAW treatment (Figure 4A) without effects on assimilatory pigments in shoots and leaves (chlorophylls and carotenoids, Figure 4B), although the average seed germination was significantly lower than the T20 treatment (Figure 2C). Also, stomatal conductance of the leaves significantly increased in PAW compared to TW, T5, and SMX (Figure 4C). As expected, the T20 treatment showed a low level of phytotoxicity for assimilatory pigments and photosynthesis, with no significant differences between controls (Figure 4B).

The absolute DW control increased the mean value of stomatal conductance in tomato seedlings by 83.7 units more than TW, while PAW had 90.99 units less. However, DW did not show significant differences of means between TW and the rest of the treatments, except PAW. In contrast, although PAW was appropriate for the absolute DW control, it significantly increased the evapotranspiration rates respecting TW, T5, and SMX irrigation solutions, suggesting a higher plant demand for the hydric regime under this condition. T20 was shown to induce similar responses to all plant treatments in terms of stomatal conductance analyses (Figure 4C). The dry root and shoot biomasses obtained in the pot-scale experiment for the absolute control (DW) did not show significant differences compared to the rest of the treatments (Figure 4D). On the other hand, the plants in the TW control had a similar root biomass as shown for PAW, T20, and T5 which were recorded with a higher dry weight compared to those in which SMX solution was used. Moreover, only the shoots irrigated with TW were significantly higher than those watered with T5 solution (Figure 4D).

Still, the toxicity of SMX in roots is clearly revealed by the significant differences in length measurements compared to the DW control in both the Petri dishes assay (Figure 3A) and the pot trial (Figure 3B). It is further reinforced by the inhibition of root growth calculations, which exceeds the minimum of 20% growth inhibition by the control (Figure 3C). The overall biomass was not affected compared to the TW solvent control in pots (Figure 4D). However, in the Petri dish assay, the total biomass of plants grown over 10 days had the lowest weight after T20 treatment (Table 2, composite sample).

### 3.3. Soil Irrigation, Physical–Chemical, and Geochemical Characterization

**Figure 5:**
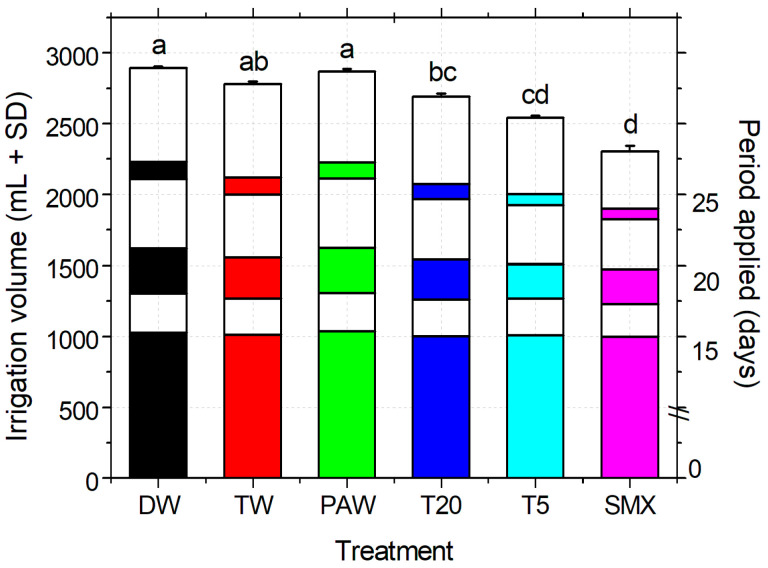
Irrigation treatments applied, volume, and timing, based on the experimental design, WHC of the selected soil, and the plant developmental stage. DW—distilled water (absolute control); TW—tap water (solvent control); PAW—plasma-activated water treatment of 20 min in plasma-ozonation system (plasma control); T20, T5—Initial SMX solution treated for 20 and 5 min respectively, using the NTP-ozonation system; SMX—untreated initial solution, 0.25 mM prepared in TW. The color of each column is correlated with the type of the solution applied for the entire paper, and the graph is plotted over six different treatment time periods, representing the total volume (+SD) of which three were treatments (days 0–15, 18–20, 25) and three DW (day 16–17, 21–24, 26–30) giving the spatial distribution of watering during the experiment. Statistical differences of means are marked with lowercase letters above the columns.

The total volume of water (DW plus treatment solutions) used for irrigation was measured throughout the experiment at the pot scale. Statistically significant differences were observed between the experimental treatments, as shown in Figure 5. There were no statistically significant differences between the control groups (DW, TW, and PAW), while T20 had comparable volumes to the solvent control (TW). At the end of the experiment, the total amount of SMX applied during irrigation was 83.76 mg L^−1^ for the initial solution and around 20 mg L^−1^ for T5 (according to the plasma degradation kinetics of the initial solution in the plasma-ozonation system [35]. Calculations of the original applied solutions, excluding DW from the total irrigation volume, showed no statistical differences between the data sets. This demonstrates that the DW does not interfere with the solvent and plasma controls, nor with the final concentration of SMX added in the media, as the plant responded accordingly to the total amount of pollutant applied.

**Figure 6:**
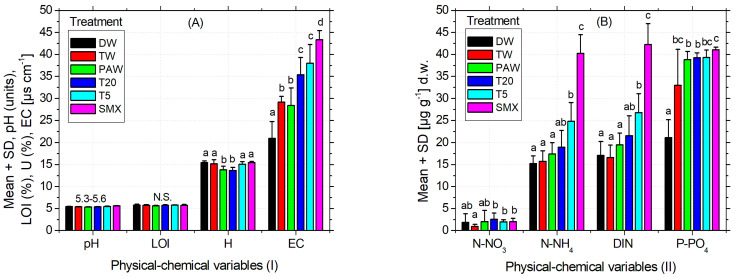
Physical–chemical analyses (**A**) (I) and (**B**) (II) of the soil after harvesting the plants. Legend: pH of the soil; EC—electrical conductivity; H—soil humidity; LOI—loss on ignition; DIN = N-NH_4_^+^ (ammonium) + N-NO_3_^−^ (nitrate) + N-NO_2_^−^ (nitrite)—dissolved inorganic nitrogen, P-PO_4_^3−^ (bioavailable phosphorus).

As can be seen in Table 3, the pH of the used soil was slightly acidic according to the INRA classification [67]. The recorded EC is weak, being specific for the medium-salted soils [68,69]. Although it is an agricultural soil, the organic carbon expressed as LOI has a low content [70] corresponding to a range from 0% to 10%. The inorganic N forms calculated as dissolved inorganic nitrogen (DIN: N-NH_4_^+^ + N-NO_3_^−^ + N-NO_2_^−^) are moderate but still good enough for the development of various plant species [71]. The content of bioavailable phosphorus is relatively low [72] as shown in Table 3. Some of the essential elements measured for plants were found acceptable, such as Fe and K (Table 3), where the optimal range for Fe is 1–3%, [73] and for K it is 0.04–3% [74]. On the other hand, Ca recorded a concentration below the recommended range of 0.3–1.0% [73], while Cu easily exceeds it (30 up to 100 mg kg^−1^), and Mn has the same pattern of excess, the maximum optimal upper limit of 525 mg kg^−1^ [73]. As the optimum values for Zn, As, Ni, and Pb are (17–125), (2–10), (30–35), and (2–60) mg kg^−1^, their concentrations were maintained within the normal values (Table 3), except for Ni, whose content is slightly higher. Overall, the selected soil for this experiment is considered suitable for a good culture [73,75]. As can be seen in Table 3, the geochemical properties of the soil, as expected, changed significantly over time for a few elements. Similarly, a part of the physical–chemical variables significantly changed after plant harvest, as shown in Figure 6A,B. Figure 6A and Table 3 indicate a slightly acidic pH and LOI that did not change significantly during the experiment or between the experimental treatments. Conversely, the moisture for the PAW treatment was significantly lower compared to the other treatments, but similar to T20. The EC recorded after plant harvest increased significantly between treatments from the left to the right (Figure 6A), with a similar pattern of variation observed for the inorganic N forms (Figure 6B). The content of bioavailable phosphorus in T20 treatment was not significantly different compared to TW and PAW but substantially different by the absolute control (Figure 6B). Overall, T20 decreased soil humidity but increased EC and P-PO_4_^3−^ compared to the absolute control (DW), while pH, LOI, N-NO_3_^−^, N-NH_4_^+^, and DIN were not significantly different from distilled water.

### 3.4. Leaf Surface Microarchitecture and Element Spectra

For the quality analyses of the plant material, six additional pots of tomato seedlings were used, grown under the same experimental conditions and optical microscopic examination of the roots, and which did not confirm the symbiosis between *Rhizophagus irregularis* and tomato seedlings under these short-time experimental conditions; thus, the extent of arbuscular mycorrhizal fungi (AMF) colonization could not be assessed.

**Figure 7:**
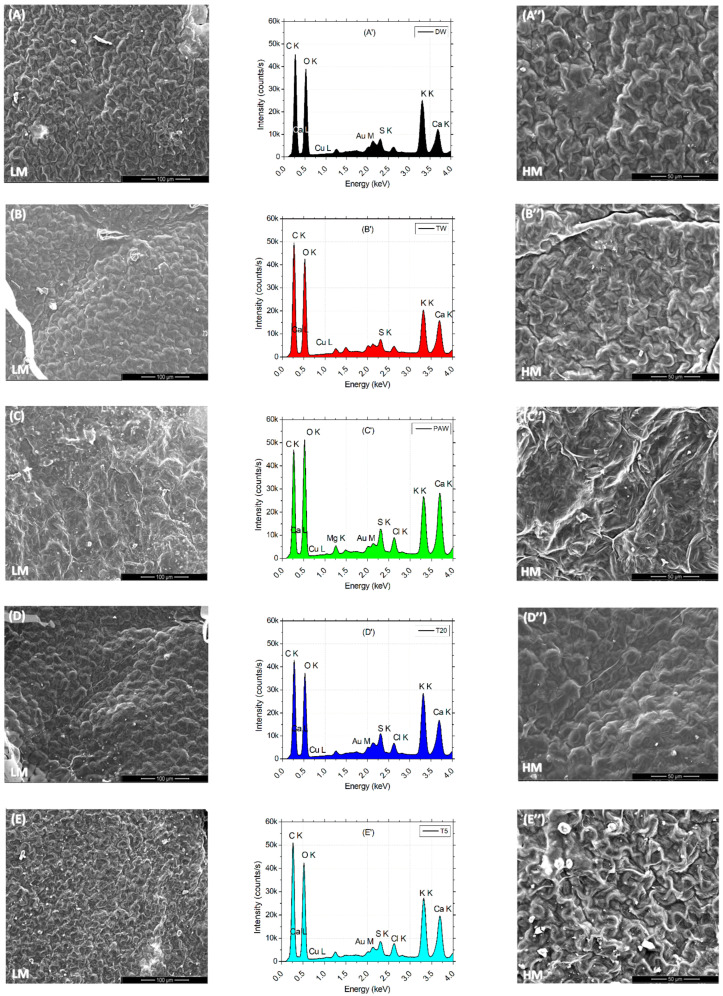

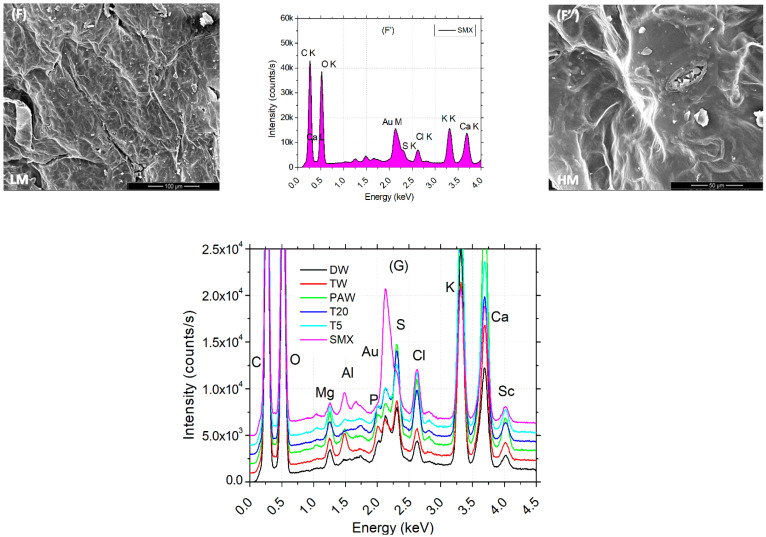
Leaf surface microarchitecture at low (**A**–**F**) and high (**A″**–**F″**) magnifications (LM and HM″) showing the presence or absence of crystals and stomata condition, associated with EDX analyses (**A′**–**F′**) for chemical elements detection for each separate treatment; (**G**)—EDS results by automatic and manual detection of elements plotted at 1000 units of distance between treatments.

Low (L) and high (H) images of the leaf surface reflect the tissue microarchitecture and state of stomata. It can be observed that the presence of the SMX concentration is correlated with the appearance of the crystal abundance on the probes from clean water (DW) to polluted water (SMX). A denser abundance can be observed in Figure 7E,E″ for T5 and also a blocked ostiole detailed in Figure 7F″ for SMX treatment. EDX analyses of leaves automatically detected the most abundant elements present in a sample from a distance of 50 μm above. Element differences between treatments were shown in separate plots and a merged spectra expanded to 1000 units of distance between treatments (Figure 7G). In the PAW sample, the element Mg was automatically detected (Figure 7C′), but not into the rest of the treatments, although still present (Figure 7G). Another particular finding was a decrease in Cu on the L line and the appearance of a high pick of Au on the M line in the SMX spectra (Figure 7F′).

## 4. Discussion

The behavior of seed germination speed, radicle emergence, and root elongation timing may vary between days and treatments at early exposure, as we observed fluctuations of until seven days between our experimental replicates. Despite the differences previously recorded in the root length observed between T5 and SMX [35], there were no statistical differences here (Figure 2A). It is clear enough that testing two different tomato cvs. (Zaraza in the present study vs. Rio Grande in the older ones [35]), using a solution with possibly lower stability due to long-term storage (4 °C), or even different growth conditions applied [35], would lead to different results. Moreover, the folate reserves in seeds can be metabolized and distributed differently also during early plant development, until the new ones are biosynthesized [76,77] after detoxification against the antifolate effects induced by SMX. In general, the above-mentioned endpoint in plants can be differently influenced depending on the type of antibiotics [78,79,80,81], the applied plasma treatment, or the plant species [35,46,82,83,84,85]. The level of MDA in SMX-treated roots suggests that cellular damage of membranes could be significantly reduced, in the detriment of leaves (Figure 4A). The auto-catalytic process involving peroxidation reactions of unsaturated lipid susceptible for degradation [86] resulted in a decreased level of MDA after SMX treatment. This biomarker for oxidative stress [66] shows an improved effect on cellular lipids in the tomato roots that could be associated with activated enzymatic defense mechanisms (i.e., SOD, CAT, GPX, etc.) under environmental water stress conditions [87,88]. However, in stems and leaves, although SMX had no significant differences by DW, photosynthesis was affected. The associated chlorophylls (a, b) and carotenoids were strongly decreased by the SMX treatment compared to the controls (Figure 4B). Although the concentration of T5 did not significantly affect the MDA content of plant, the assimilatory pigments were decreased, thus generally showing biological significance in pharmaceutical pollution related to incomplete water treatment [35,89,90,91]. Next, the water vapor fluxes measured from the leaf surface to the atmosphere [52] indicated increased stomatal conductance in tomato seedlings irrigated with PAW compared to TW. Figure 4C shows the heterogeneity of plants over such a wide range of data obtained yet correlated with the levels of assimilatory pigments found in aboveground parts (Figure 4B).

The irrigation method applied (Figure 5) allowed the timely provision of the volumes necessary for the plant growth when the plasma treatment of SMX was limited by the need for such large amounts [35]. There were no statistically significantly differences between the two data sets of the irrigation solutions with or without DW addition. These are reflected at the end of the experiment in the total volumes applied to plant requirements and daily soil wetting according to the WHC index. Overall, the responses of the seedling growth were induced only by the real uptake of the SMX concentrations accumulated in the media [16] and by the partial treatment of plasma-derived SMX solutions (T5) based on their morphometric analyses. In our view, this can be considered a suitable irrigation method for similar laboratory experimental conditions if the present restrictive advanced oxidation process technology is used (330 mL for every treatment time). Thus, extending the experimental duration with the aim of obtaining more data of the stage of plant flowering and fruit formation would mostly require large volumes of water.

Alternating irrigation days between treated solutions and DW could be a viable approach. However, the experimental design could be improved by including an additional harvest of seedlings from the pots originally planted with 12 seeds each, as biomass tends to increase over time. This will not only provide space for further plant development, avoiding root competition for food and surface crowding for light, but will also allow additional time for the preparation of the plasma solutions. Moreover, it is preferable to harvest the seedling in time until one or, better, two plants remain in a single pot. This will allow the exploration of other defense mechanisms during the exposure, which were not possible to analyze in this test due to the lack of enough biomass.

The recorded pH of the soil that was determined to have a medium acidity could affect the crop fertility [58], while its specific *EC* is related to medium-salted soils [68,69]. In general, EC can be influenced by the ion movement in the solvent (i.e., temperature, viscosity), its physical properties (i.e., size, +/− charge or concentration), but also by the extract composition (Figure 6A). Regarding the soil humidity (Figure 6A) and the total irrigation volume applied (Figure 5) as a result of plant water demands, these could be discussed in the PAW and T20 treatments, which were significantly lower compared to all the other treatments. Similar patterns of variation also appear in the forms of inorganic N by increasing the quantity of the drug-derived elements present in the media (Figure 6B). The content of bioavailable phosphorus was not significantly different between TW and PAW treatments but significantly different from the absolute control (Figure 6B). As the recorded Mn exceeded the upper optimal limit, this may be associated with parental rock composition and origin of the Romanian soils, which are predominantly higher in this element (Figure 6B).

The EDS automatically detected an excess of Mg ions in the PAW treatment (Figure 7C′), which generally constitutes the central element of the chlorophyll’s porphyrin ring [86], while the SEM images of leaf microarchitecture captured some opened stomata, which is a good qualitative indicator of the physiological state of the plant in plasma applications [35,41,46,92]. The automatic EDX detection of the Mg element in the PAW spectrum by the EDS system (Figure 7C′) could explain the increased concentration of chlorophylls a and b (Figure 4B). Although the highest column of PAW graphs does not show statistical differences between any other control referred, the content of carotenoids recorded in the TW was much lower than in the PAW treatment. This could further be related to the optimal evapotranspiration process compared to TW (Figure 4C), as the SEM images (Figure 7C,C″) display the absence of SMX-derived crystals. The water vapor flux measured with the foliar porometer provides clue information about the state of the stomata such as density, size, and opening degree, being a good indicator of the plant physiology status. Since the stomata’s sensitivity is influenced by the light intensity, humidity, CO_2_ concentration, water stress, pathogens, and pollution [66,86], the evapotranspiration rate could also be negatively affected. The ostiole condition (Figure 7F″) associated with water stress regime in a polluted environment, such as SMX, may play a role in aquaporin regulation [93]. Interestingly, the pick levels of carbon and oxygen within the leaf surface, seem to remain constant for each treatment alone (except for the counting intensity), while for PAW, the oxygen turns out to be higher than the carbon.

The same trends are observed for the pick elements K and Ca, where for the DW, TW, T5, and SMX treatments (Figure 7A′,B′,E′,F′) they showed similarities (except for the intensity), while the PAW could influence the Ca uptake by the plants [94]. The data merged by artificially adding 1000 units of distance between the treatments allowed to visualize the ensemble of picks and the differences in the pattern between DW, TW, PAW, T20, T5, and SMX treatments for each element with excess concentration (Figure 7G).

## 5. Conclusions

The seed germination in the Petri dish test was significantly improved by the undegraded SMX solution compared to DW and PAW. This response was similar to that in the TW treatment, but root length was shorter compared to the rest of the samples. On the other hand, the T20 irrigation treatment resulted in a similar increase in the final percentage of seed germination to the control solutions, while root growth was promoted. The root length in T20 plates was clearly greater compared to roots in the absolute DW control, expressing more appropriate characteristics to PAW (plasma) and TW (solvent) controls. On the other hand, in the pot assay T20 induced less than 20% (NS) root inhibition by DW in soil and improved some morphological, physiological, and biochemical variables in tomato seedlings. The specific results of these experiments led to the conclusion that further investigations of the same type are needed on various other plant species. Future investigations will focus on the ecophysiology of species (fauna and flora) that could exist in agricultural ecosystems where the application of reused water would be possible. A great interest would also be broader analyses that would include experimental food products generated under such conditions. The expansion of studies on such products would ensure key answers for sustainable marketing, human nutrition status, and human health risks based on future modern agriculture.

## Figures and Tables

**Table 1:** pH and EC variables of the solutions used in all experimental treatments. Legend: DW—distilled water; TW—tap water; PAW—plasma-activated water treatment for 20 min in plasma-ozonation system; T20, T5—initial SMX solution treated for 20 and 5 min, respectively, using the NTP-ozonation system; SMX—untreated initial solution, 0.25 mM dissolved in TW.

Variable	DW	TW	PAW	T20	T5	SMX
pH units, 21 °C	6.570	7.664	8.362	7.406	7.463	7.366
EC μS cm^−1^, 21 °C	2.5	330	334	335	326	324

**Table 2:** Biomass of tomato seedlings in Petri dish, composite samples. Legend: DW-distilled water; TW-tap water; PAW-plasma-activated water; T20-SMX solution treated with NTP for 20 min; T5 = SMX solution treated with NTP for 5 min; SMX = sulfamethoxazole.

*Solanum lycopersicum* L. cv Zaraza							
Day 10	Unit	DW	TW	PAW	T20	T5	SMX
Root biomass	mg per plant d.w.	0.62	0.62	0.65	0.65	0.48	0.46
Shoot biomass		1.15	0.67	1.54	0.45	1.45	1.7
Total weight		1.77	1.29	2.19	1.1	1.93	2.16

**Table 3:** Soil physical–chemical and geochemical variables (*N* = 5). Legend: pH of the soil; EC—electrical conductivity; H—soil humidity; LOI—loss on ignition; DIN = N-NH_4_^+^ (ammonium) + N-NO_3_^−^ (nitrate) + N-NO_2_^−^ (nitrite)—dissolved inorganic nitrogen, P-PO_4_^3−^ (bioavailable phosphorus). Statistical differences are marked with lower case letters above the columns.

Physical–chemical variables before plant sowing									
	pH	EC	H	LOI	N-NH_4_^+^	N-NO_3_^−^	N-NO_2_^−^	DIN	P-PO_4_^3−^
Scale	Units	[µs cm^−1^]	[%]		[µg g^−1^] dry soil				
Mean	5.27	53.6	4.94	6.2	17.3	9.3	0.022	26.7	9.8
SD	0.03	5.238	0.15	1.03	1.6	2.1	0.003	2.7	0.07
Geochemical variables (mg kg^−1^) before seeding the plants									
Element	Ca	Cu	Fe	K	Mn	Zn	As	Ni	Pb
Mean initial	2516 a	39.5 a	21,600 a	10,488 a	791.5 a	71.2 a	9.2 ab	61.7 a	17.3 a
*SD*	*527.4*	*8*	*529.1*	*1476*	*58.94*	*2.9*	*2.4*	*17.3*	*2.3*
Treatments	Geochemical variables (mg kg^−1^) after plant harvesting								
DW	2114 ab	32.1 ab	19,546 b	10,164 a	665.4 c	61.3 b	8 ab	43.7 a	15.5 ab
TW	1717 b	28.8 b	19,551 b	9258 a	671 bc	63.3 cb	8.2 ab	46.9 a	12.9 bc
PAW	2379 a	29.8 ab	20,173 c	8443 a	719.5 ab	68.1 ac	8.7 a	53.7 a	15.7 ab
T20	2257 ab	31.4 ab	20,124 c	9743 a	705.1 bc	63.3 bc	7.8 ab	57.4 a	13.3 bc
T5	2571 a	33.2 ab	19,620 bc	8604 a	700.2 bc	64 bc	7.4 b	54.9 a	14.7 abc
SMX	2449 a	28.8 ab	19,715 bc	9534 a	687.7 bc	61.5 b	8.4 ab	58.4 a	12 c

## Data Availability

Data are available upon request to the first author.
